# Charge Injection and Auger Recombination Modulation for Efficient and Stable Quasi‐2D Perovskite Light‐Emitting Diodes

**DOI:** 10.1002/advs.202309500

**Published:** 2024-03-06

**Authors:** Kwan Ho Ngai, Xinwen Sun, Xinhui Zou, Kezhou Fan, Qi Wei, Mingjie Li, Shiang Li, Xinhui Lu, Weiwei Meng, Bo Wu, Guofu Zhou, Mingzhu Long, Jianbin Xu

**Affiliations:** ^1^ South China Academy of Advanced Optoelectronics South China Normal University Guangzhou 510006 China; ^2^ Department of Electronic Engineering The Chinese University of Hong Kong Shatin New Territories 999077 Hong Kong; ^3^ Department of Physics and William Mong Institute of Nano Science and Technology The Hong Kong University of Science and Technology Clear Water Bay Kowloon 999077 Hong Kong; ^4^ Department of Applied Physics The Hong Kong Polytechnic University Kowloon 999077 Hong Kong; ^5^ Department of Physics The Chinese University of Hong Kong Shatin New Territories 999077 Hong Kong

**Keywords:** ion migration, low‐dimensional perovskite, operational lifetime, perovskite light‐emitting diodes, recombination dynamics

## Abstract

The inefficient charge transport and large exciton binding energy of quasi‐2D perovskites pose challenges to the emission efficiency and roll‐off issues for perovskite light‐emitting diodes (PeLEDs) despite excellent stability compared to 3D counterparts. Herein, alkyldiammonium cations with different molecular sizes, namely 1,4‐butanediamine (BDA), 1,6‐hexanediamine (HDA) and 1,8‐octanediamine (ODA), are employed into quasi‐2D perovskites, to simultaneously modulate the injection efficiency and recombination dynamics. The size increase of the bulky cation leads to increased excitonic recombination and also larger Auger recombination rate. Besides, the larger size assists the formation of randomly distributed 2D perovskite nanoplates, which results in less efficient injection and deteriorates the electroluminescent performance. Moderate exciton binding energy, suppressed 2D phases and balanced carrier injection of HDA‐based PeLEDs contribute to a peak external quantum efficiency of 21.9%, among the highest in quasi‐2D perovskite based near‐infrared devices. Besides, the HDA‐PeLED shows an ultralong operational half‐lifetime *T*_50_ up to 479 h at 20 mA cm^‒2^, and sustains the initial performance after a record‐level 30 000 cycles of ON–OFF switching, attributed to the suppressed migration of iodide anions into adjacent layers and the electrochemical reaction in HDA‐PeLEDs. This work provides a potential direction of cation design for efficient and stable quasi‐2D‐PeLEDs.

## Graphical abstract

The chain length tuning of alkyldiammonium cations in quasi‐two dimensional perovskites is utilized to modulate the recombination dynamics and injection efficiency of perovskite light‐emitting diodes. A moderate exciton binding energy, efficient energy transfer and balanced carrier injection in quasi‐two dimensional perovskite based on cation with appropriate length contribute to an external quantum efficiency of 21.9% and reduced roll‐off issue.

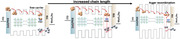

## 1 Introduction

Organic–inorganic hybrid perovskites have been widely researched as the light emitters in next‐generation display technology, owning to the extraordinary optoelectronic properties, such as high photoluminescence quantum yield (PLQY), high bidirectional charge carrier mobility, narrow emission spectrum, and tunable emission color.^[^ ^1^, ^2^, ^3^, ^4^, ^5^, ^6^, ^7^, ^8^, ^9^, ^10^ ^]^ Besides, perovskite light‐emitting diodes (PeLEDs) are well adaptable to printable fabrication process due to the feature of facile solution processability.^[^ ^11^, ^12^ ^]^ Recently, with continuous efforts on perovskite composition, device structure, processing techniques, and defect passivation, the performance of PeLEDs has obtained impressively rapid improvement and the external quantum efficiency (EQE) has been comparable to the commercialized light‐emitting didoes technology.^[^ ^12^, ^13^, ^14^, ^15^, ^16^ ^]^ However, the PeLEDs suffer from serious ion migration issue under electric field, resulting in electrochemical reaction between adjacent layers and perovskite decomposition.^[^ ^17^, ^18^ ^]^ Bulky organic cations with various geometries have been introduced into 3D perovskites to form quasi‐two‐dimensional (quasi‐2D) perovskites, consisting of alternative inorganic and organic layers, which aims to increase the ion migration barrier and suppress the decomposition process.^[^ ^19^, ^20^, ^21^ ^]^

The quasi‐2D perovskite films typically consist of quasi‐2D phases with a series of different inorganic layers and also some 3D phases. The charges or excitons in the quasi‐2D phases can naturally funnel to 3D phase with a smaller bandgap, which serves as the recombination centers in the perovskite layer. The ultrafast funneling process can potentially outcompete trap‐assisted nonradiative recombination process, and thus it is favorable for the light‐emission efficiency.^[^ ^22^, ^23^, ^24^ ^]^ Besides, the distinct dielectric constant difference between the organic ligand and inorganic framework in quasi‐2D perovskites results in quantum confinement effect. The strongly bound excitons dominate the excitonic recombination process, which enhance the quantum yield and lead to significant improvement in luminous efficiency.^[^ ^25^ ^]^ Highly efficient PeLEDs based on the quasi‐2D perovskite light‐emitting layer with different organic interlayers, have been extensively demonstrated, attributing to the efficient excitonic recombination and the cascading energy‐transfer process between different phases.^[^ ^26^, ^27^, ^28^, ^29^, ^30^ ^]^ However, despite the merits of high photoluminescence yield of quasi‐2D perovskites, the ultrafast exciton/carrier transfer process from small‐*n* phases to large‐*n* ones (*n* = ∞) leads to a surge of carrier density in 3D perovskite, which would inevitably increase the probability of Auger recombination and thus result in serious roll‐off issue.^[^ ^31^, ^32^, ^33^ ^]^ Besides, the strongly bound excitons in quasi‐2D perovskite with quantum confinement structure would also accelerate the Auger recombination rate, which further decreases the radiative recombination efficiency at large current density, leading to energy loss and low emission efficiency for quasi‐2D PeLEDs under high carrier density. The configuration design and size modulation of organic cation are effective methods to modulate the exciton binding energy, energy transfer process, and the Auger recombination rate in quasi‐2D perovskites, leading to enhanced photoluminescence yield over a wide range of carrier density.^[^ ^34^, ^35^, ^36^ ^]^ However, although the chain length tuning of organic cations has been considered as an intuitive approach to tune the recombination dynamics in 2D perovskite‐based photovoltaic devices, it has rarely been investigated in the corresponding light‐emitting devices.^[^ ^37^, ^38^ ^]^ Another consideration is that the incorporation of large cations would decrease the electrical conductivity of the perovskite layer and thus lowering the carrier injection efficiency, which is directly related with the chain size of the cations.^[^ ^22^, ^39^, ^40^, ^41^ ^]^ Therefore, chain length tuning of large cations in quasi‐2D perovskite can be utilized to simultaneously modulate the radiative recombination rates and the injection barrier in quasi‐2D perovskite emissive layer, aiming to reduce the Auger recombination and optimize luminous efficiency of the PeLEDs.

Here, the alkyldiammonium cations with different molecular sizes, namely 1,4‐butanediamine (BDA), 1,6‐hexanediamine (HDA) and 1,8‐octanediamine (ODA), are employed into quasi‐2D perovskites, to simultaneously tune the carrier injection efficiency and exciton binding energy. The size increase of the bulky cation leads to increased exciton binding energy from 108.6, 138.1 to 168.6 meV for the *n* = 5 BDA, HDA, and ODA‐based quasi‐2D perovskites, respectively, which leads to a gradually increased excitonic recombination rate. However, carrier dynamics show that the Auger recombination rate is inevitably increased with the increase of molecule size, leading to more serious roll‐off issue of the corresponding PeLEDs. Besides, larger bulky cation assists in the formation of randomly distributed 2D perovskite nanoplates, which results in less efficient injection and deteriorates the electroluminescence (EL) performance. Finally, a moderate exciton binding energy, suppressed 2D phases and balanced carrier injection of the HDA‐based PeLEDs contribute to a peak external quantum efficiency (EQE) of 21.9%, among the highest in quasi‐2D perovskite‐based near‐infrared devices. Besides, the HDA‐PeLED shows an ultralong operational half‐lifetime *T*_50_ of 479 h at 20 mA cm^‒2^, and sustains the initial performance after 30 000 continuous cycles of ON‐OFF switching, attributed to the suppressed migration of iodide anions into adjacent layers and the electrochemical reaction for the organic cations. This work suggests that delicate organic cation design provides further solution to the efficiency and stability of PeLEDs.

## 2 Results and Discussion

### 2.1 Electroluminescent Performances

**Figure 1:**
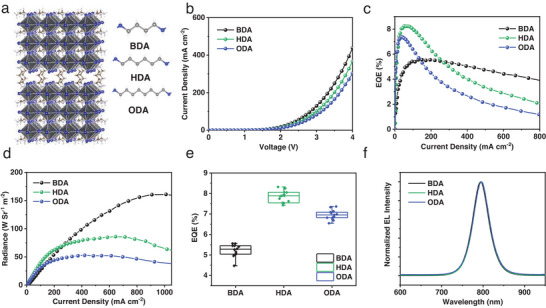
EL performance of the DJ PeLEDs. a) Chemical structures of BDA, HDA, ODA, and the typical DJ perovskite. b) Current density‐voltage curves, c) EQE–current density curves, d) radiance–current density curves, e) EQE statistics (12 devices for each), and f) normalized EL spectra of DJ PeLEDs with different ligand chain lengths.

The perovskite precursor solutions are prepared by mixing the reactants FAI and PbI_2_ with different alkyldiammonium iodide, namely BDAI_2_, HDAI_2_, and ODAI_2_, according to the chemical stoichiometry of quasi‐2D Dion‐Jacobson (DJ) perovskite LFA_4_Pb_5_I_16_, where L denotes the long chain organic cation. The structures of the alkylammonium molecules and the typical DJ perovskite are displayed in **Figure** **1**a. The PeLEDs were fabricated through spin‐coating process with a typical structure of tin‐doped indium oxide (ITO)/ZnO/polyethylenimine ethoxylated (PEIE)/perovskite/poly[(9,9‐dioctylfluorenyl‐2,7‐diyl)‐co‐(4,4′‐(*N*‐(p‐butylphenyl))diphenylamine)] (TFB)/MoO_3_/Au as illustrated in Figure S1 (Supporting Information). The current density–voltage (*J–V*) characteristics of the PeLEDs with different chain lengths are compared in Figure 1b. It is obviously shown that the current density is gradually decreased with the increase of carbon length, which results from the increased carrier transport barrier of alkyldiammonium cation. As shown in Figure 1c, the peak EQE of BDA‐PeLED is 5.5%, largely increased to the highest value of 8.2% for HDA‐PeLED and slightly decreased to 7.3% for ODA‐PeLED. The overall poor EL performance could be attributed to the deficient FA cation owning to the deprotonation process on ZnO surface.^[^ ^42^, ^43^ ^]^ It is noted that the current density corresponding to the peak EQE values is decreased from 171.5 mA cm^−2^ for BDA‐, 115.3 mA cm^−2^ for HDA‐, and significantly to only 46.1 mA cm^−2^ for ODA‐PeLEDs, respectively. It results from more intense quantum confinement effect and efficient exciton recombination process in the quasi‐2D perovskite with longer chain length, which suppress the trap‐assisted nonradiative recombination process with low current injection.^[^ ^25^, ^34^ ^]^ Meanwhile, it is obviously demonstrated that the roll‐off issue becomes more serious with the increase of chain length.

Even though the EQE values are increased for the HDA‐ and ODA‐based devices as compared to the BDA‐one, the radiance decreases significantly from 160.9 W Sr^−2^ m^−2^ for BDA‐PeLED to 85.9 W Sr^−2^ m^−2^ and 52.8 W Sr^−2^ m^−2^ for HDA‐ and ODA‐PeLEDs, respectively, as displayed in Figure 1d, which can be correlated with the decreased injection carrier density. Both the EQE and radiance statistics are summarized in Figure 1e and Figure S2 (Supporting Information), suggesting the highest EQE value for HDA‐PeLEDs and a reliable reproducibility of PeLEDs based on quasi‐2D perovskites. All the EL peaks show no appreciable shift and are located at ≈794 nm with a narrow full width at half maximum of 45 nm as illustrated in Figure 1f, which indicates the large‐*n* (*n* = ∞) phase serving as the emitting centers in the LFA_4_Pb_5_I_16_ perovskites as confirmed by the PL spectra in Figure S3 (Supporting Information).

### 2.2 Properties of DJ Perovskite Thin Films

**Figure 2:**
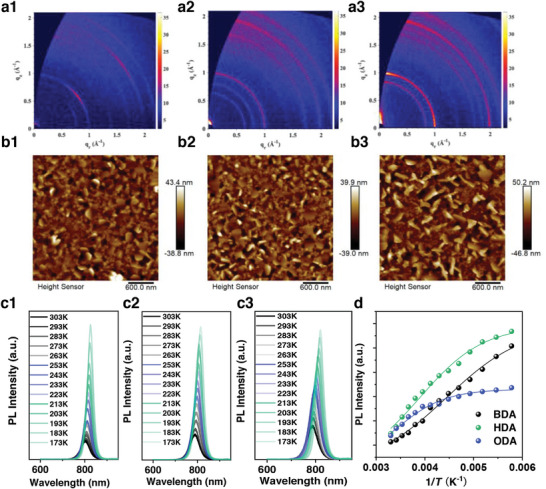
Properties of DJ perovskite films. a1–a3) GIWAXS patterns of BDA‐, HDA‐ and ODA‐based DJ perovskite films. b1–b3) AFM images of BDA‐, HDA‐, and ODA‐based DJ perovskite films (areal size of 3 × 3 µm). c1–c3) Temperature‐dependent PL spectra of BDA‐, HDA‐, and ODA‐DJ perovskite films. d) PL intensity and the fitting curves as a function of temperature for the DJ perovskite films.

The crystallinity, phase distribution and film morphology of the LFA_4_Pb_5_I_16_ DJ perovskite thin films are systemically investigated to study the effect of carbon chain length. Diffraction peaks with low intensity located at 8.3° and 10.3°, 7.7° and 9.0° are observed for the BDA‐ and HDA‐DJ perovskites, respectively, as shown in the X‐ray diffraction (XRD) patterns of the *n* = 5 DJ perovskite films in Figure S4 (Supporting Information), indicating the formation of quasi‐2D DJ phases. However, the diffraction intensity of these peaks is relatively low, reflecting a random crystal orientation of the quasi‐2D perovskite films. There is not any diffraction peak in the small‐angle range for the ODA‐based perovskite film, while the excitonic absorption peaks are obviously observed as shown in the UV‐Vis absorption spectra in Figure S5 (Supporting Information). The absence of diffraction peaks for the quasi‐2D phases in the ODA‐DJ perovskite could be attributed to random orientation of the small‐*n* phase due to the larger formation energy of the 2D phases with longer chain length.^[^ ^44^ ^]^ Besides, two diffraction peaks located at 11.7° and 13.9° are observed for all the DJ perovskite films suggesting the existence of both *δ*‐ and *α*‐FAPbI_3_ phases. The peak intensity of *α*‐FAPbI_3_ is gradually increased with the increase of chain length, which could be attributed to the enhanced templating effect of the alkyldiammonium molecules with larger size.^[^ ^45^ ^]^ Grazing incidence wide‐angle X‐ray scattering (GIWAXS) characterization is further employed to study the crystal structure of the DJ perovskite films, and the results are displayed in **Figure** **2**a1–a3 and Figure S6 (Supporting Information). All the GIWAXS patterns exhibit a typical Bragg ring at *q*_z_ = 1 Å, suggesting a random crystal orientation. However, the diffraction intensity is gradually increased with elongated chain length. Diffraction rings located at *q* = 0.76 Å for BDA‐DJ, *q* = 0.64 Å and *q* = 0.79 Å for HDA‐DJ perovskites, respectively, indicate the formation of quasi‐2D species. There is not any quasi‐2D phase observed in the GIWAXS patterns for the ODA‐based perovskite, which is consistent with the XRD results.

Furthermore, the film morphology of the DJ perovskites is characterized through atomic force microscopy (AFM) as displayed in Figure 2b1–b3. Regular plate‐shaped crystals with size ≈200 nm are observed in all the samples, which correspond to the quasi‐2D phases as indicated from the GIWAXS and absorption spectra. The plate‐shaped crystals become more obvious in the perovskite with the increase of chain length. However, these nanoplates are tilted and randomly distributed on the film surface, which can account for the low diffraction intensity in the BDA‐ and HDA‐based perovskites, and the absence of diffraction peaks of quasi‐2D phases in ODA‐based perovskite. The random orientation and the enhanced composition of quasi‐2D perovskites could be responsible for the reduced current density and poor EL performances for the ODA‐PeLEDs.

To investigate the excitonic properties of the alkyldiammonium cation‐based DJ perovskite films with different chain length, temperature (*T*)‐dependent photoluminescence (PL) with *T* ranging from 173 K to 373 K is applied to track the peak and intensity tendency as shown in Figure 2c1–c3,d. All the PL intensity increases with decreased temperature, which is more obvious in the low‐*T* range. The peak position shows a gradually blue shift with the increase of *T*, which is due to the thermal expansion of the crystal structure and reduced orbital overlaps.^[^ ^46^ ^]^ From the integrated area of PL spectra as a function of the reciprocal of temperature shown in Figure 2d, the binding energy (*E*_b_) is extracted with the equation as follow:*I*(*T*) = *I*_0_/(1 + *B*exp ( − *E_b_*/(*k_B_T*)), where *k*_B_ is the Boltzmann constant and *B* is the pre‐exponential factor.^[^ ^47^ ^]^ The *E*_b_ values of BDA‐, HDA‐, and ODA‐DJ perovskite films are estimated to be 108.6, 138.1, and 168.6 meV, respectively. The increased *E*_b_ value leads to stronger quantum confinement effect and also larger Auger recombination rate, which is responsible for the more serious roll‐off phenomenon in the ODA‐PeLEDs. Furthermore, the transient absorption (TA) spectra of the quasi‐2D perovskites (*n* = 5) with different cations are further compared in Figure S7 (Supporting Information) to elucidate the impact of cation chain length on the phase distribution and energy transfer process. The photoinduced changes in the TA spectra demonstrate that the BDA‐, HDA‐ and ODA‐DJ perovskites possess the same phase compositions as indicated from the similar absorption peaks displayed in Figure S7a–c (Supporting Information). The peak intensity for the large‐*n* (*n* = ∞) phase in the ODA‐DJ perovskite is remarkably weaker as compared to the BDA‐ and HDA‐ones, which suggests the inefficient energy transfer from small‐*n* phases to large‐*n* one despite the enhanced crystallinity. The large‐*n* phase in BDA‐DJ perovskite shows a much slower carrier accumulation with a building‐up time of 0.37 ps as indicated from the TA decay kinetics in Figure S7d (Supporting Information). The photobleaching peak for the large‐*n* phase in HDA‐DJ perovskite reaches a maximum amplitude with an ultrafast time of 0.17 ps, suggesting the more efficient exciton transfer as shown in Figure S7e (Supporting Information), which is advantageous to the radiative recombination in the large‐*n* perovskite. Moreover, the ODA‐DJ perovskite demonstrates a slower exciton transfer even between small‐*n* phases as shown in Figure S7f (Supporting Information), which can be attributed to the larger transfer barrier owing to the increased chain length. Therefore, the fast exciton transfer and strongly bound carriers in the HDA‐PeLEDs result in higher possibility to outcompete the trap‐assisted recombination process, which is responsible for the high EQE value at a low current injection.

### 2.3 Quantum Confinement in the DJ Perovskites

**Figure 3:**
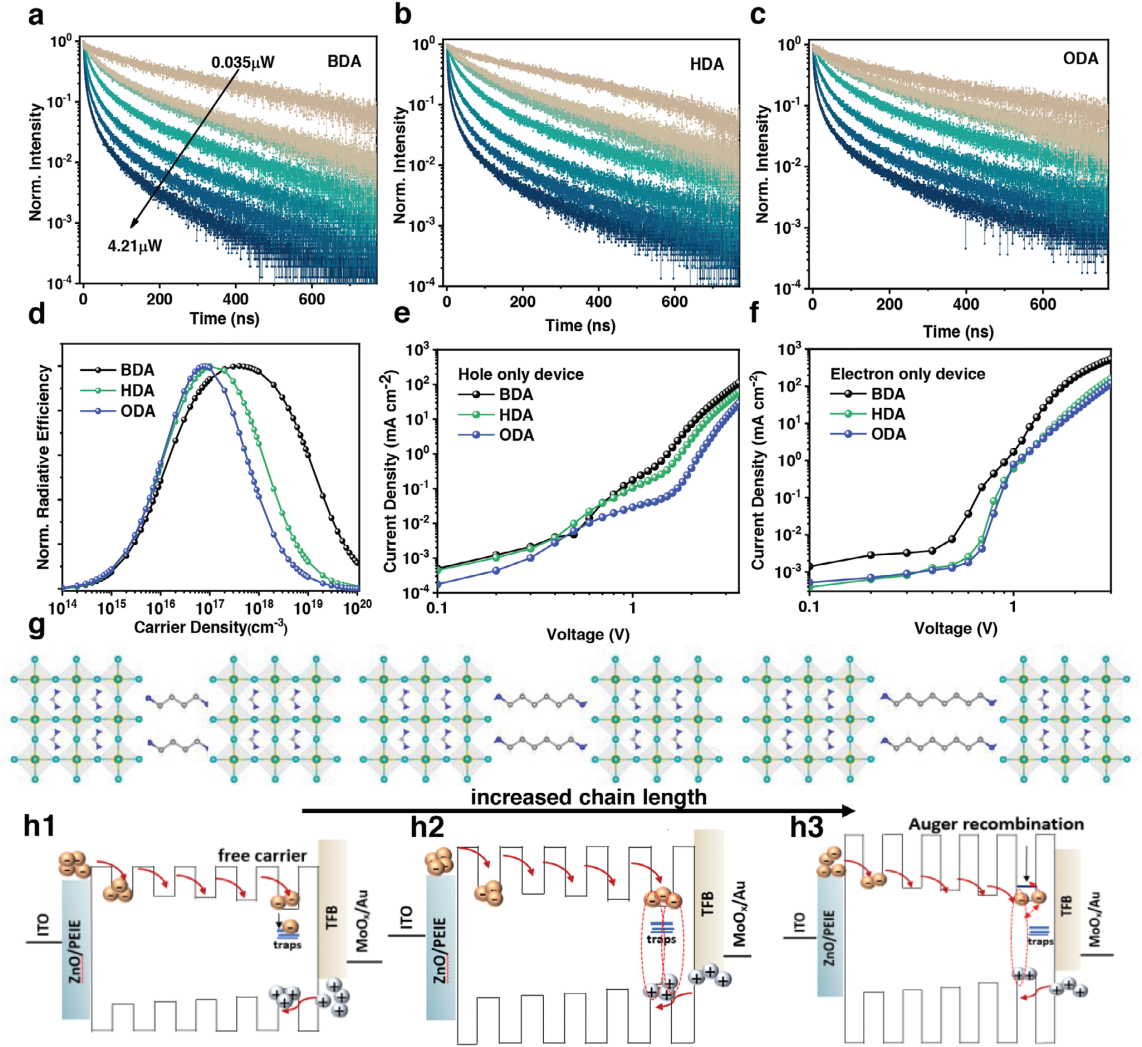
Quantum confinement effect in DJ perovskites. Power‐dependent TRPL spectra for a) BDA‐, b) HDA‐, and c) ODA‐perovskite films. d) Simulated radiative efficiency based on fixed *k*_1_ of 5 × 10^6^ s^−1^ and extracted *k*_2_, *k*_3_ values. Current density–voltage curves of e) hole‐only devices and f) electron‐only devices with different perovskite layers. g) The crystal structure of BDA‐, HDA‐, and ODA‐perovskites. h1–h3) Schematic diagram of the carrier recombination dynamics in BDA‐, HDA‐, and ODA‐PeLEDs.

To further understand the recombination dynamics of those DJ perovskites, power dependent time‐resolved photoluminescence (TRPL) with a laser power ranging from 0.035 to 4.21 µW is further employed to compare the monomolecular, bimolecular, and trimolecular recombination processes in the DJ films as shown in **Figure** **3**a–c and Table S1 (Supporting Information). To avoid carrier accumulation after excitation, each sample requires a cool‐down process before testing. The carrier dynamics can be described by the equation as follows: *dn*/*dt* = −*k*_1_ *n* − *k*_2_ *n* ^2^ − *k*_3_ *n* ^3^.^[^ ^48^ ^]^ With low excitation intensity, the TRPL curves follow a single‐exponential decay, which is attributed to the dominant first‐order recombination process. The monomolecular recombination constants *k_1_* extracted from the curves with low pump fluence are 6.71 × 10^6^, 5.08 × 10^6^, and 4.04 × 10^6^ s^−1^ for BDA‐, HDA‐ and ODA‐DJ perovskites, respectively, as shown in Table S1 (Supporting Information). As the monomolecular recombination constant *k*_1_ depends on both the trap‐assisted nonradiative recombination and exciton recombination, the tendency of the exciton recombination constant cannot be quantitatively obtained here. To eliminate the effect of trap‐assisted nonradiative recombination, the normalized radiative efficiencies (*ϕ*) with fixed *k_1_* value (5 × 10^6^ s^−1^) are calculated by the equation as follows: $\phi \left(n\right)=\frac{nk_{2}}{k_{1}+nk_{2}+n^{2}k_{3}}$. The tendency of radiative efficiency as shown in Figure 3d correlates well with the trend of maximum EQE in Figure 1c. With the increase of ligand length, the peak position shifts from higher charge‐carrier density to much lower values, confirming that the excitonic recombination is more significant in lower injection region for the ODA‐PeLED. Further global fitting reveals that the trimolecular Auger recombination constant, *k*_3_, is increased from 4.34×10^−29^ s^−1^ cm^6^ for BDA‐DJ perovskite, to 3.62×10^−28^ s^−1^ cm^6^ and 6.59×10^−28^ s^−1^ cm^6^ for HDA‐ and ODA‐DJ perovskites, respectively. The one‐order larger *k*_3_ for the ODA‐perovskite as compared to the BDA‐one can be attributed to the larger *E*_b_ and increased quantum confinement, which contributes to the more serious roll‐off issue of the corresponding PeLED.

In addition, the charge injection efficiency of the PeLEDs is characterized by applying the DJ perovskite films into hole‐only devices (ITO/PEDOT:PSS/perovskite/TFB/Au) and electron‐only devices (ITO/ZnO/PEIE/perovskite/PCBM/Au). The dark *J*‐*V* curves summarized in Figure 3e,f demonstrate that higher current density can be achieved by perovskite films with shorter ligand chain length in both the electron‐ and hole‐only devices, which is attributed to the increased injection barrier of the DJ perovskite with longer chain ligand. Therefore, the poor performance of the ODA‐based PeLED is ascribed not only to the serious Auger recombination, but also to the decreased electron and hole injection efficiency. The appropriate exciton binding energy and injection efficiency lead to the enhanced EL performance for the HDA‐PeLEDs. A schematic diagram of the impact of different cations in quasi‐2D perovskite is provided to highlight the key impacts of different cations in quasi‐2D perovskites on the injection barrier and recombination dynamics as shown in Figure 3g,h1–h3. Specially, the smaller exciton binding energy in BDA‐DJ perovskite leads to slower recombination process and increased trap‐assisted recombination rates as displayed in Figure 3h1.^[^ ^48^ ^]^ However, the increased chain length of alkyldiammonium cations in perovskites would increase the injection barriers from the carrier transport layer to the perovskite and also from the inorganic slabs to the organic cations in the perovskite as displayed in Figure 3h2,h3. The largest size of ODA cation inevitably leads to the reduced current density of the corresponding devices. The enhanced confinement of the excitons in inorganic slabs increases the Auger recombination rates, which is responsible for the serious roll‐off issue and lower EQE at high current density. Therefore, the compromise between exciton recombination rate and injection efficiency in HDA‐DJ perovskite makes it as a more appropriate light‐emitting material.

### 2.4 Performance Optimization and Stability Evaluation

**Figure 4:**
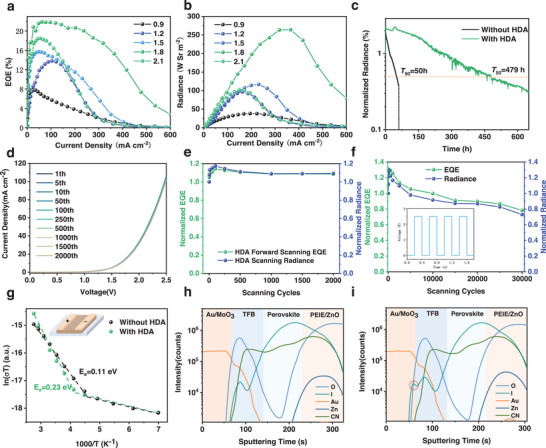
Performance optimization and stability properties. a) EQE‐current density and b) radiance–current density curves of the HDA‐based PeLEDs with different FAI to PbI_2_ ratios. c) Operational lifetime measurement for 1.8FA PeLED with/without HDA. d) Current density‐voltage curves, and e) EQE and radiance tendencies as a function of ramping cycle for 1.8FA HDA PeLED under continuous forward‐bias scanning. f) EQE and radiance tendencies under continuous ON‐OFF switching with a 2.5 Hz, 2.5 V pulse for 1.8FA HDA PeLEDs. g) Extraction of the activation energy for ion migration from the temperature‐dependent conductivity measurement of the perovskites with/without HDA. Depth‐dependent ion distribution for the h) fresh and i) degraded 1.8FA PeLEDs without HDA.

To further optimize the carrier injection efficiency and suppress the trap‐assisted nonradiative recombination of the HDA‐PeLEDs, further composition tuning of the small cations and the incorporation of passivation agent are employed to improve the EL performance of HDA‐PeLEDs. Here, the devices based on perovskites with FAI to PbI_2_ molar ratios *x* ranging from 0.9 to 2.1 are abbreviated as *x*FA HDA‐PeLED for simplicity. Besides, passivation additive 2‐(2‐(2‐aminoethoxy)ethoxy)acetic acid (AEAA) is incorporated into precursor solution to suppress traps and improve optoelectronic properties.^[^ ^49^ ^]^ The EQE and radiance tendency of the *x*FA HDA‐PeLED are summarized in **Figure** **4**a,b. It is demonstrated that the EQE values are gradually increased from 7.9% to 13.8% and 15.7% for the 0.9FA, 1.2FA, and 1.5FA PeLEDs, respectively, and to the highest value of 21.9% for the 1.8FA device, and further decreased to 18.4% for 2.1FA HDA‐PeLED. The radiance has a similar trend as the EQE, with the value increasing from 38.2, 96.6, 116.7 W Sr^−1^ m^−2^ for 0.9FA, 1.2FA, 1.5FA HDA‐PeLEDs, respectively, to the highest value of 142.4 W Sr^−1^ m^−2^ for the 1.8FA one, and then decreasing to 101.9 W Sr^−1^ m^−2^ for 2.1FA HDA‐PeLEDs. The current density increases first but then decreases significantly with the increase of FA ratio as shown in Figure S8 (Supporting Information), which indicates that the excess FA cation is not beneficial to the current injection. The optimized EL performance through FA ratio tuning can be attributed to the enhanced crystallization and also the enhanced optoelectronic property as shown in Figure S9 (Supporting Information). Besides, the perovskite crystal size is enlarged with the addition of excess FA as compared in Figure S10 (Supporting Information). However, with the FA/Pb ratio further increased to 2.1, the crystal domains become less uniformly dispersed and the crystal size is also more uneven as compared to the 1.8FA one. The 1.8FA HDA perovskite with more uniformly distributed crystal domains and uniform crystal size can be beneficial for improving the light‐outcoupling efficiency.^[^ ^6^ ^]^

The effect of HDA incorporation on the EL performance and operational stability of the PeLEDs is further compared as shown in Figure S11 (Supporting Information) and Figure 4c. The 1.8FA PeLED without HDA shows an EQE value of 18.2% and a radiance of 124.7 W Sr^−1^ m^−2^, which are inferior to the device with HDA incorporation. For the operational stability, the radiance of the PeLEDs is continuously monitored under a constant current density of 20 mA cm^−2^, and the time of the radiance drops to 50% of the initial value is considered as half‐lifetime (*T*_50_). Both the devices with or without HDA show a radiance overshoot at the very beginning stage, which can be attributed to the ion migration and trap healing process under carrier injection.^[^ ^50^, ^51^ ^]^ The 1.8FA HDA‐PeLED shows an extremely longer *T*_50_ of 479 h as compared to the control device of only 50 h. It is worth noting that the radiance fluctuation under constant current can be attributed to the interfacial ion and charge accumulation of the device under electric field.^[^ ^52^ ^]^ Moreover, the stability of the devices is further investigated through continuous voltage scanning from 0 to 2.5 V with a step of 0.1 V. The current density–voltage curves, and EQE and radiance tendencies for those devices under ramping are summarized in Figure 4d,e and Figure S12a,b (Supporting Information), respectively. Although the current density of both devices with or without HDA shows a negligible change during the entire scanning, both the EQE and radiance of the 1.8FA PeLED drop to 75% of its initial values after only 500 cycles. The1.8FA HDA‐PeLED can maintain almost its initial EQE and radiance after 2000 scanning cycles, ensuring the reliable stability under electric field. Furthermore, the ON–OFF switching endurance for those devices are further evaluated by a 2.5 V, 2.5 Hz pulse voltage as shown in Figure 4f and Figure S12c (Supporting Information). Both EQE and radiance drop to 70% of the initial values with only 700 switching cycles for 1.8FA PeLED, while the 1.8FA HDA‐PeLED can sustain ≈80% of the initial values after 30 000 cycles. The sudden current injection could initiate serious ion migration in perovskite, which corresponds to the perovskite decomposition and performance degradation for the PeLEDs without HDA.

To further understand the degradation mechanism of the PeLEDs under electric field, a lateral conduction configuration is prepared for temperature‐dependent voltage‐current measurement with the structure shown as the inset in Figure 4g. The corresponding voltage‐current curves with temperature ranging from 143 to 363 K are summarized in Figure S13 (Supporting Information), showing a gradually increased current for both 1.8FA perovskite films with or without HDA with the increase of temperature. The relationship between temperature and conductivity of the perovskite layers is plotted in Figure 4g. Obviously, both the curves show a transition temperature from electronic conductor to ionic conductor, which are 213 K for 1.8FA perovskite and 243 K for HDA 1.8FA HDA‐perovskite, indicating suppressed ion migration process after HDA incorporation.^[^ ^53^ ^]^ Furthermore, the relationship between conductivity and activation energy (*E*_a_) for ion migration can be described by the Nernst–Einstein equation as follows: σ(*T*) = (σ_0_/*T*)exp ( − *E_a_*/*k_B_T*), where *σ*_0_ is the pre‐exponential factor.^[^ ^53^ ^]^ The *E*_a_ is extracted from the ionic dominant region, and *E*_a_ of the 1.8FA perovskite films without or with HDA are estimated to be 0.11 and 0.23 eV, respectively. Therefore, the dominant origin of the poor operational stability for the reference device is the accelerated ion migration under electric field. The twofold larger *E*_a_ for the HDA‐perovskite films is responsible for the lifetime optimization under long‐term bias poling.^[^ ^54^ ^]^

Furthermore, to understand the ion migration effect on the decomposition manner after long‐term electric poling, the ion distribution comparison between fresh and degraded devices is characterized by time‐of‐flight secondary ion mass spectrometry (ToF‐SIMS) depth profiling as illustrated in Figure 4h,i and Figure S14 (Supporting Information). In general, organic cation FA^+^ and anion I^−^ are considered as the main components of ion migration.^[^ ^55^, ^56^ ^]^ It is well noted that iodide diffusion and accumulation toward the anode can be observed in ZnO tranporting layer in both fresh PeLEDs with or without HDA as displayed in Figure 4h and Figure S14a (Supporting Information), suggesting the inevitably mild halide‐diffusion across interfaces even without electric bias. It can be attributed to the deprotonation process of FA cations on ZnO surface, leading to the interfacial reaction between ZnO and iodide ions.^[^ ^42^ ^]^ After long‐term voltage stress, the degraded PeLED without HDA shows further iodide infiltration toward Au/MoO_3_ and obviously increased accumulation at the interface as indicated by the red circle in Figure 4i, while the iodide composition does not show any obvious change for the degraded HDA‐PeLED shown in Figure S14b (Supporting Information). Furthermore, the composition of the molecular fragment CN in the degraded device without HDA shows significant reduction, indicating the accelerated deprotonation of FA^+^ owing to the electrochemical reaction between FA^+^ and ZnO under electric field as shown in Figure S15a (Supporting Information). On the contrary, there is a negligible change of the CN component in the ZnO layer for the HDA‐PeLED as shown in Figure S15b (Supporting Information). There is not any impurity observed despite the decreased PL intensity and crystallinity of the perovskite‐emitting layer in 1.8FA PeLED without HDA after performance degradation as shown in Figures S16,S17 (Supporting Information). The PL intensity and the main diffraction peak in the XRD pattern of the perovskite in 1.8FA HDA‐PeLED are well maintained, further confirming the enhanced operational stability. Therefore, the iodide migration toward the metal anode and the deprotonation of FA^+^ in ZnO layer are significantly suppressed in the device after HDA incorporation, which account for the remarkably improved stability under long‐term constant electric stress and pulse bias.

## 3 Conclusion

In summary, alkyldiammonium cations with different chain lengths are incorporated into quasi‐2D DJ perovskites to simultaneously tune the carrier recombination dynamics and carrier injection efficiency. The increased exciton recombination in the long‐chain cation‐based DJ PeLEDs leads to accelerated excitonic radiative recombination and also increased Auger recombination rate. Besides, the larger size of the alkyldiammonium cation leads to less efficient injection owning to the insulating nature, and thus deteriorating the EL performance. A moderate exciton binding energy, suppressed 2D phases and balanced carrier injection of the HDA‐based PeLEDs contribute to a peak EQE value of 21.9%, which is among the highest in quasi‐2D perovskite‐based near‐infrared devices. Furthermore, the HDA‐PeLED demonstrates an ultralong *T*_50_ of 479 h at 20 mA cm^‒2^, and sustains 80% of the initial performance after 30 000 cycles of ON‐OFF switching. The ToF‐SIMS results further demonstrate that the suppressed migration of iodide anions into metal electrode and reduced deprotonation of FA^+^ in the ZnO layer are responsible for the remarkably enhanced stability of HDA‐PeLEDs. This work indicates that the bulky cation design can be further extended to optimize the performance and also operational stability of quasi‐2D‐PeLEDs.

## 4 Experimental Section

### Materials

Formamidinium iodide (FAI; ≥99%, anhydrous) was purchased from Merck. 1,4‐butanediamine iodide (BDAI_2_), 1,6‐hexanediamine iodide (HDAI_2_), and 1,8‐octanediamine iodide (ODAI_2_) were acquired from Xi'An Polymer LightTechnology Corp. Lead iodide (PbI_2_; 99.999%) was procured from Tokyo Chemical Industry. Ethanolamine (99%) was purchased from Thermo Fisher Scientific Inc. Polyethylenimine 80% ethoxylated solution (PEIE) was purchased from Sigma‐Aldrich. Poly[(9,9‐dioctylfluorenyl‐2,7‐diyl)‐co‐(4,4′‐(*N*‐(p‐butylphenyl))diphenylamine) (TFB, molecular weight: 10 000–200 000) was obtained from American Dye Source. All the organic solvents were procured from Sigma‐Aldrich unless specifically stated.

### PeLED Device Fabrication

The etched ITO substrates with size of 12×12 mm^2^ were cleaned sequentially with Hellmanex aqueous solution, deionized water, acetone, isopropanol, and ethanol for 15 min each. Subsequently, the substrates were treated with ultraviolet‐ozone for 30 min to enhance the surface wettability. The ZnO nanocrystals in ethanol solution were synthesized using a simple precipitation method in accordance with the previous report.^[^ ^42^ ^]^ The ZnO layer was spin‐coated on the substrate at 5000 r.p.m. for 40 s, followed by annealing at 150 °C for 30 min. After cooling down, a PEIE solution (3.9 mg mL^−1^ in 2‐methoxyethanol) was spin‐coated onto the ZnO surface at 5000 r.m.p. for 30 s, followed by annealing at 100 °C for 10 min. Then, the substrates were transferred to the N_2_ filled glovebox for depositing the remaining layers. The perovskite precursor with a composition of LFA_4_Pb_5_I_16_ (L = BDA, HDA, ODA) was dissolved in *N, N*′‐dimethylformamide (DMF) solvent for phase pure *n* = 5 DJ perovskites. For optimized HDA perovskite precursor, excess FAI and passivation agent 2‐(2‐(2‐aminoethoxy)ethoxy)acetic acid (AEAA) were added into the previous solution with molar ratio of AEAA to Pb of 0.5. The perovskite precursor was spin‐coated at 6000 r.p.m., followed by annealing at 100 °C for 15 min. After cooling down to room temperature, the TFB solution (15 mg mL^‒1^ in m‐xylene) was spin‐coated onto perovskite at 3000 r.p.m. Finally, a sequential deposition of 7 nm MoO*_x_* and 50 nm Au was carried out in a thermal evaporation chamber under high vacuum, using a shadow mask to define the emission area of 2.25 mm^2^_._

### Characterization

The current density–voltage–radiance (*J–V–R*) curves were characterized and collected by a Keithley 2400 Source Meter and a fiber optic integrating sphere coupled with a QE65 Pro spectrometer (Ocean Optics). The devices were characterized on top of the integrating sphere which collects the radiance from the bottom of the transparent ITO substrates in N_2_ glovebox. The absolute radiance intensity was calibrated by a standard vis–NIR light source (HL‐3P‐INT‐CAL, Ocean Optics). GIWAXS characterization  was conducted at 23 A SWAXS beamline, using a 10 keV primary beam, 0.1° incident angle, Pilatus 1M‐F, and C9728 detector. UV–vis absorption spectra were measured by a Hitachi U‐3501 UV–vis–NIR spectrophotometer. The XRD patterns of the thin films were carried out by a Rigaku SmartLab X‐ray diffractometer equipped with Cu K_α_ (*λ* = 1.54 Å) and a HyPix‐3000 2D hybrid pixel array detector. AFM images of perovskite film were taken with a Bruker's Dimension Icon AFM in standard tapping mode at room temperature. The surface morphology was characterized by JSM‐7800F SEM microscope. The transient dynamics of the perovskite was acquired by Helios setup, and a nondegenerate pump–probe configuration was applied to probe the transient data. A collinear optical parametric amplifier (Coherent OPerA Solo) generated the pump by a regenerative amplifier (Coherent Legend Elite system, 800 nm). A Ti‐sapphire oscillator (Coherent Vitesse, 80 MHz, 100 fs) was equipped to seed the amplifier. Steady‐state PL spectra were measured by commercially available Raman/PL spectrometer (Horica, Inc.) with an air‐cooled double frequency Nd: Yag 532 nm laser source. Time‐resolved PL spectra were obtained by Horiba Instruments spectrometer (iHR320). The excitation source was a picosecond pulsed laser (DD‐470L) with a pulse width of below 100 ps and a repetition rate of 1 MHz. Element analysis of perovskite films was carried out by ION ToF SIMS 5–100.

## Conflict of Interest

The authors declare no conflict of interest.

## Supporting information

Supporting Information

## Data Availability

The data that support the findings of this study are available from the corresponding author upon reasonable request.
