# Supplementary material for: Charge Injection and Auger Recombination Modulation for Efficient and Stable Quasi‐2D Perovskite Light‐Emitting Diodes
Source: Adv Sci (Weinh). 2024 Mar 6;11(18):2309500. doi: 10.1002/advs.202309500 (PMC11095209; doi:10.1002/advs.202309500)
Supplement: Supplementary file 1 — Supporting Information [file ADVS-11-2309500-s001.pdf]

ADVANCED SCIENCE
Open Access

Supporting Information

for *Adv. Sci.*, DOI 10.1002/advs.202309500

Charge Injection and Auger Recombination Modulation for Efficient and Stable Quasi-2D Perovskite Light-Emitting Diodes

Kwan Ho Ngai, Xinwen Sun, Xinhui Zou, Kezhou Fan, Qi Wei, Mingjie Li, Shiang Li, Xinhui Lu, Weiwei Meng, Bo Wu, Guofu Zhou, Mingzhu Long and Jianbin Xu**

Supplementary Information

Charge injection and Auger recombination modulation for efficient and stable quasi-2D perovskite light-emitting diodes

Kwan Ho Ngai[1,2,†], Xinwen Sun[2,†], Xinhui Zou[3], Kezhou Fan[3], Qi Wei[4], Mingjie Li[4], Shiang Li[5], Xinhui Lu[5], Weiwei Meng[1], Bo Wu[1], Guofu Zhou[1], Mingzhu Long[1,*], Jianbin Xu[2,*]

Affiliations

[1] South China Academy of Advanced Optoelectronics, South China Normal University, Guangzhou, 510006, China.

[2] Department of Electronic Engineering, The Chinese University of Hong Kong, Shatin, New Territories, 999077, Hong Kong.

[3] Department of Physics and William Mong Institute of Nano Science and Technology, The Hong Kong University of Science and Technology, Clear Water Bay, Kowloon, 999077, Hong Kong.

[4] Department of Applied Physics, The Hong Kong Polytechnic University, Kowloon, 999077, Hong Kong.

[5] Department of Physics, The Chinese University of Hong Kong, Shatin, New Territories, 999077, Hong Kong.

†E-mail: mzlong@m.scnu.edu.cn, jbxu@ee.cuhk.edu.hk

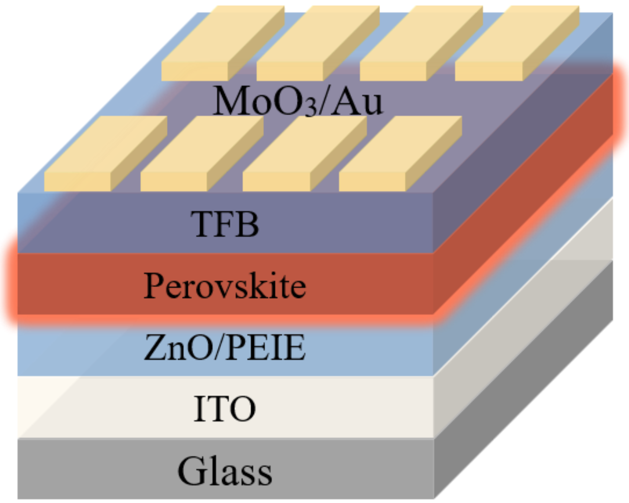

Figure S1. Device architecture of PeLED.

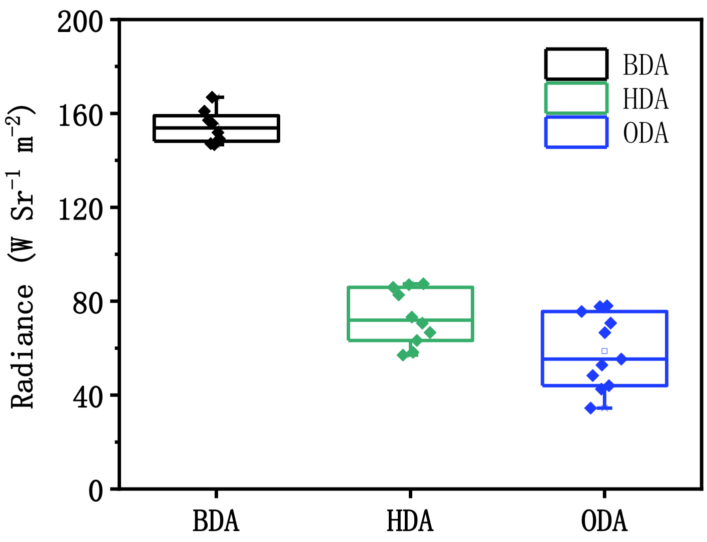

Figure S2. Radiance statistics of DJ PeLEDs with different ligand chain lengths.

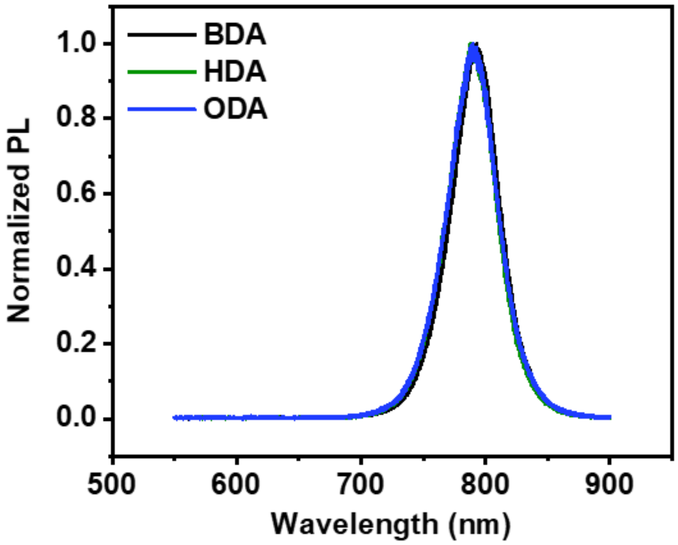

Figure S3. PL spectra of the BDA-, HDA-, and ODA-based DJ perovskite ($LFA_4Pb_5I_{16}$) films.

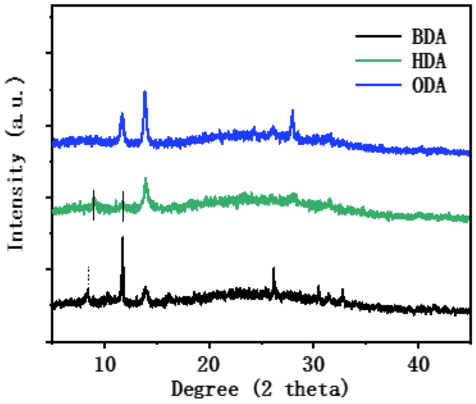

Figure S4. XRD patterns of the BDA-, HDA-, and ODA-based DJ perovskite ($LFA_4Pb_5I_{16}$) films.

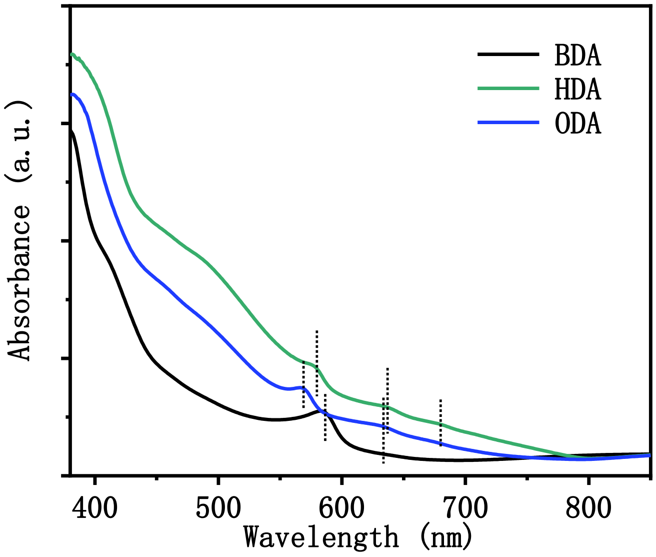

Figure S5. UV-vis absorption spectra of the n = 5 BDA-, HDA- and ODA-DJ perovskite films.

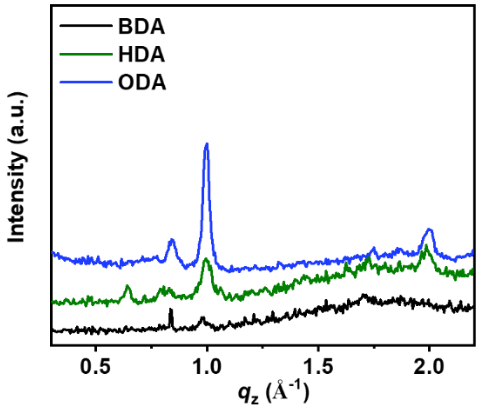

Figure S6. Corresponding azimuthally integrated intensity profiles obtained from the GIWAXS patterns along the q_z axis.

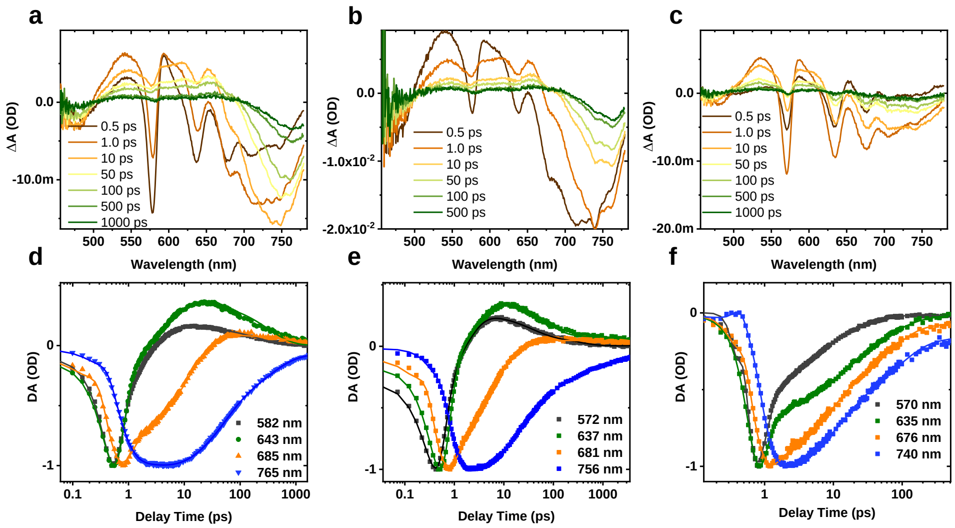

Figure S7. (a-c) TA spectra of BDA-, HDA- and ODA-DJ thin films (n = 5). (d-f) Normalized bleaching kinetics at different wavelengths (excitation wavelength of 400 nm and excitation intensity of 1.1 μJ cm^{-2}).

Table S1. Monomolecular (k_1), bimolecular (k_2) and trimolecular (k_3) recombination constant extrated from the power dependent TRPL curves in Figure 4a-c.

	k_1 (s^{-1})	k_2 (s^{-1} cm^3)	k_3 (s^{-1} cm^6)
BDA	6.71×10^{6}	5.75×10^{-10}	4.34×10^{-29}
HDA	5.08×10^{6}	4.44×10^{-10}	3.62×10^{-28}
ODA	4.04×10^{6}	2.98×10^{-10}	6.59×10^{-28}

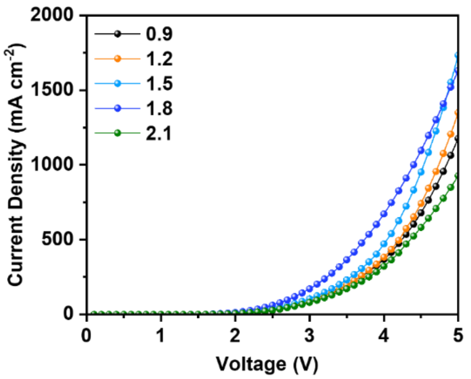

Figure S8. Current density-voltage curves of the HDA-based PeLEDs with different FAI to PbI_2 ratios.

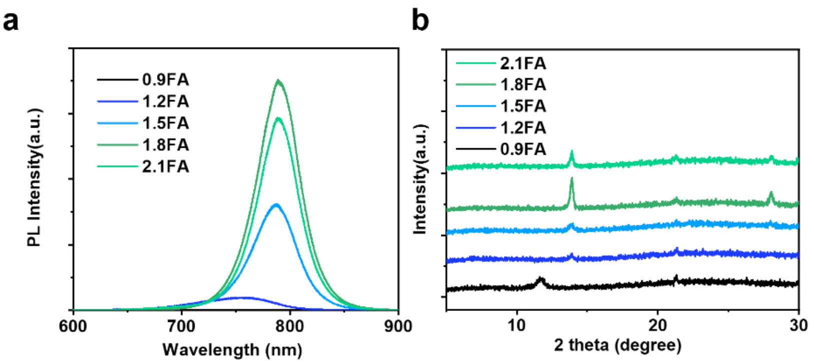

Figure S9. (a) PL spectra and (b) XRD patterns of HDA-DJ perovskite with different FA ratios.

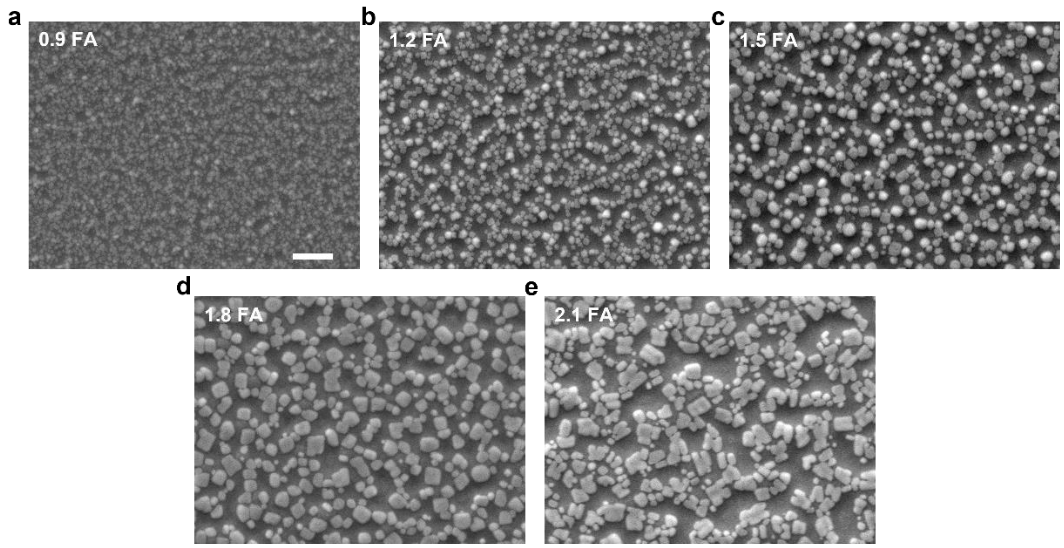

Figure S10. SEM images of *x*FA HDA perovskite films with *x* (the molar ratio of FA to Pb in the precursor) = 0.9, 1.2, 1.5, 1.8 and 2.1 (scale bar of 1 μm).

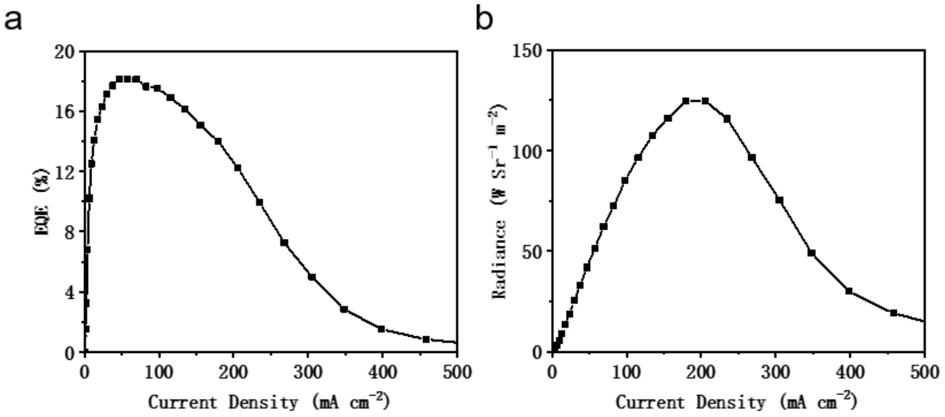

Figure S11. (a) Current density-voltage curve and (b) radiance-voltage curve of 1.8FA PeLED without HDA.

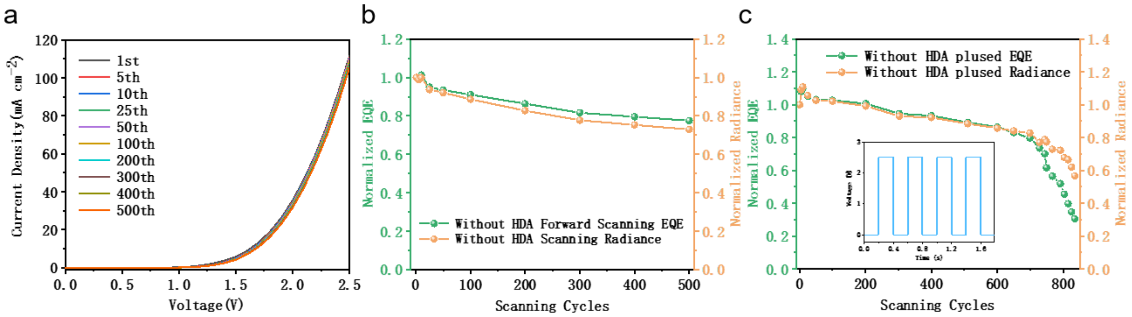

Figure S12. (a) Current density-voltage curves and (b) EQE and radiance tendency as a function of scanning cycle for 1.8FA PeLEDs without HDA under continuous forward bias scanning. (c) EQE and radiance under continuous ON-OFF switching for 1.8FA PeLEDs without HDA.

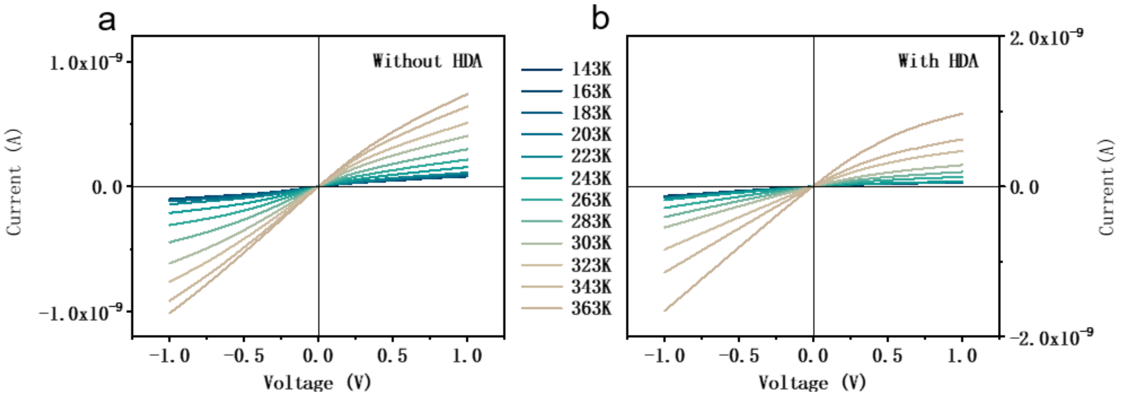

Figure S13. Temperature dependent current-voltage curves with temperature ranging from 143 K to 363 K.

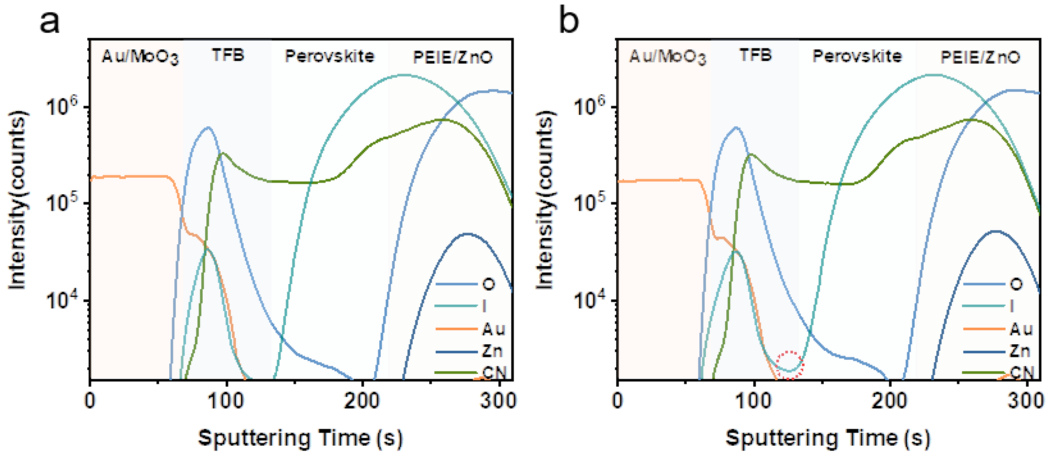

Figure S14. Ion distribution from the (a) fresh and (b) degraded 1.8FA PeLEDs with HDA.

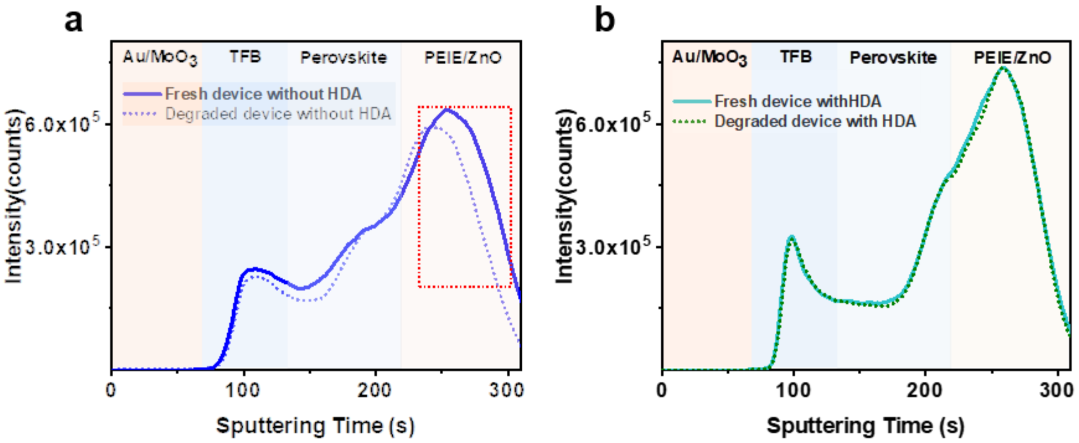

Figure S15. The in-depth distribution of CN fragment in fresh and degraded PeLEDs without (a) or with (b) HDA incorporation.

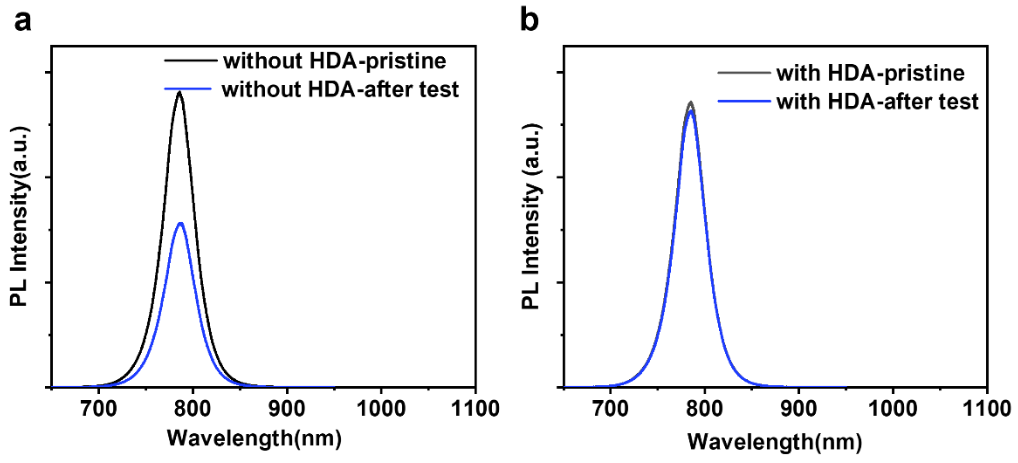

Figure S16. PL spectra of perovskite light-emitting area of the PeLEDs without (a) and with (b) HDA before and after performance degradation.

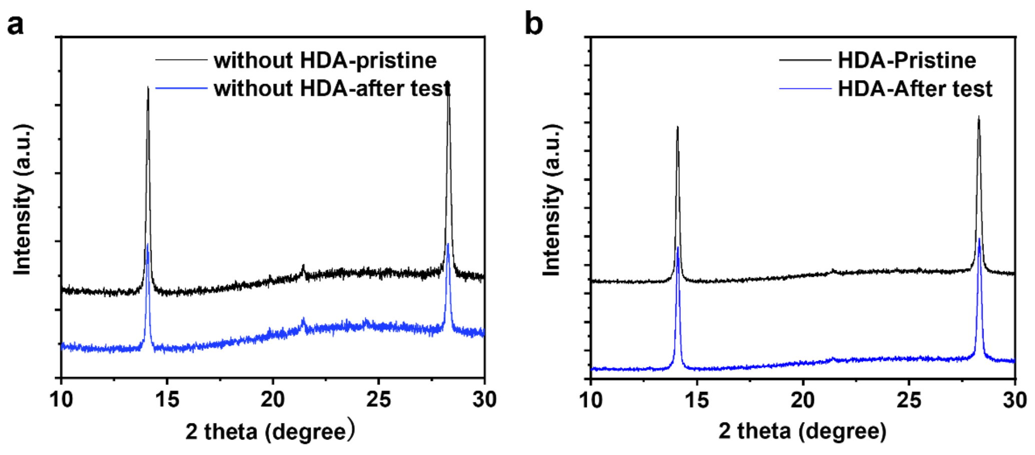

Figure S17. XRD patterns of perovskite light-emitting area of the PeLEDs without (a) and with (b) HDA before and after performance degradation.
